# A computational strategy for estimation of mean using optimal imputation in presence of missing observation

**DOI:** 10.1038/s41598-024-57264-y

**Published:** 2024-03-18

**Authors:** Subhash Kumar Yadav, Gajendra K. Vishwakarma, Dinesh K. Sharma

**Affiliations:** 1 Department of Statistics, Babasaheb Bhimrao Ambedkar University, Lucknow, 226025 India; 2 Department of Mathematics and Computing, Indian Institute of Technology (ISM) Dhanbad, Dhanbad, 826004 India; 3 Department of Business, Management and Accounting, University of Maryland Eastern Shore, Princess Anne, MD 21853 USA

**Keywords:** Estimation, Imputation, Missing data, Medical research, Mathematics and computing

## Abstract

In this study, we suggest an optimal imputation strategy for the elevated estimation of the population mean of the primary variable utilizing the known auxiliary parameters for the missing observations. Under this strategy, we suggest a new modified Searls type estimator, and we study its sampling properties, mainly bias and mean squared error (MSE), for an approximation of order one. The introduced estimator is compared theoretically with the estimators of population mean in competition under the imputation method. The efficiency conditions for the introduced estimator to be more efficient than the estimators in the competition are derived. To be sure about the efficiencies, these efficiency conditions are verified through the three natural populations. We have also conducted a simulation study and generated an artificial population with the same parameters as a natural population. The estimator with minimum MSE and the highest Percentage Relative Efficiency (PRE) is recommended for practical use in different areas of applications.

## Introduction

The arithmetic mean is the most accurate way to measure central tendency when the population being studied is homogeneous for the characteristic being studied. Because of time and financial restrictions, estimating the arithmetic mean is crucial for large populations for determining various policy decisions and other uses. These days, missing data or non-response is a prevalent and unavoidable problem and is a prevalent problem with data received from sampling surveys. These missing numbers make data analysis, processing, and handling more complex. Missing data is a concern in clinical or life-saving drug testing trials since some experimental units have to be eliminated during the experiment. Similar to this, during agricultural experiments, crops are destroyed by disease or other natural disasters. For a variety of reasons, responses from every unit in the sample are typically not available in demographic and socioeconomic surveys. This kind of incompleteness is referred to as non-response, and judgments about the population parameters may be tainted if the necessary information regarding the nature of non-response is not present.

For a long time, sample survey professionals have known that failing to account for the random character of incompleteness or non-response might degrade the data quality. In surveys, there are two forms of non-responses: unit non-response and item non-response. When an eligible sample unit is entirely absent from the survey, unit non-response occurs. Missing values can be replaced by imputations and then treated as any other auxiliary variable. However, this depends on the way the imputations are derived. It is well known that in sampling designs the use of auxiliary information improves the precision of an estimator substantially for instance see Vishwakarma and Kumar^1^ and Kumar and Vishwakarma^2^ and Vishwakarma et al.^3^. In contrast, item non-response occurs when a sampled unit is present in the survey but fails to provide information about a component of a unit in the sample survey. Missing data is an issue in such cases. Numerous studies adopted the imputation method to address this issue, which involves substituting values for missing data. It is a highly suggested method for resolving non-response issues in sample surveys, Singh et al.^4^.

Rubin^5^ suggested three methods for missing observations in survey sampling, namely, Missing At Random (MAR), Observed At Random (OAR), and Parameter Distinctness (PD). The difference between MAR and Missing Completely at Random (MCAR) was discussed by Heitjan and Basu^6^. Various authors, including, Singh and Vishwakarma^7^, Kadilar and Cingi^8^, Diana and Perri^9^, Gira^10^, Bhushan and Pandey^11^, Prasad^12^, Audu et al.^13^, Audu et al.^14^, Singh et al.^4^ and others worked on the MCAR technique and suggested different imputation methods for efficient estimation of population mean of the main variable under the situation of missing observations. The theory of sample surveys was expanded by Shahzad et al.^15^ to include imputation-based mean estimators in the event of missing data, using variance–covariance matrices and robust regression. Shahzad et al.^16^ proposed estimators of the quantile regression-ratio type for estimating means under both partial and complete auxiliary data. In order to achieve enhanced population mean estimate, Alomair and Shahzad^17^ worked on compromised imputation and EWMA based memory-type mean estimators employing quantile regression. Lawson^18^ presented a novel approach to imputation for estimating population mean in survey sampling when there are missing data. Robust quantile regression was used by Anas et al.^19^ to develop compromised imputation-based mean estimators for better population mean estimation.

Let $U = (U_{1} ,\,U_{2} ,...,U_{N} )$ be the finite population under consideration consisting of $N$ distinct and identifiable units. Further, let $Y$ be the main characteristic under investigation with a population mean as $\overline{Y} = \frac{1}{N}\sum\limits_{i = 1}^{N} {Y_{i} }$ and population variance as $\sigma^{2} = \frac{1}{N}\sum\limits_{i = 1}^{N} {(Y_{i} - \overline{Y})^{2} }$. To estimate $\overline{Y}$, we draw a random sample $s$ of size $n$ using Simple Random Sampling Without Replacement (SRSWOR). Let $r$ be the responding units belongs to $R$, the set of all responding units and $n - r$ be the non-responding units belongs to $R^{c}$, the set of all non-responding or missing units out of $n$ sampled units from the above population of size $N$. For every *i*th unit belonging to $R$ that is $i \in R$, the corresponding value of $y_{i}$ is observed, while for $i \in R^{c}$, the value of $y_{i}$ is missing and is estimated through different imputation methods. For elevated estimation of $\overline{Y}$ under imputation methods, many authors utilized the known population mean ($\overline{X}$) of the auxiliary variable $x$. Let $x_{i}$ be the observation for the *i*th unit of $x$ and is positive for all $i \in s$. Now let $y_{.i}$ be the observation on $Y$ such that:

$$y_{.i} = \left\{ \begin{gathered} y_{i} \,\,\,\,{\text{if}}\,\,i \in R \hfill \\ \tilde{y}_{i} \,\,\,\,{\text{if }}i \in R^{c} \hfill \\ \end{gathered} \right.$$

where, $\tilde{y}_{i}$ is the imputed value for the *i*th non-responding unit and by the utilization of above data, the point estimator of $\overline{Y}$ under the imputation method is given by:

$$t = \frac{1}{n}\sum\limits_{i = 1}^{n} {y_{.i} } = \frac{1}{n}\left[ {\sum\limits_{i \in R} {y_{i} } + \sum\limits_{{i \in R^{c} }} {\tilde{y}_{i} } } \right]$$

In the present study, we also suggested a new imputation method for elevated estimation of $\overline{Y}$ using the known auxiliary parameters under MCAR mechanism. We study the large sampling properties of the suggested estimator for the first order of approximation. The conditions of efficiency for the proposed estimator over the estimators in competition are derived and are verified using the three natural populations along with one simulated population. The most efficient estimator is recommended for practical use in different areas of applications.

## Review of imputation estimators

The most appropriate imputation estimator for estimating $\overline{Y}$ is obtained through the following imputation method as:

$$y_{.i} = \left\{ \begin{gathered} y_{i} \,\,\,\,{\text{if}}\,\,i \in R \hfill \\ \tilde{y}_{i} \,\,\,\,{\text{if }}i \in R^{c} \hfill \\ \end{gathered} \right.$$

and the resultant point estimator of $\overline{Y}$ is,

$$t_{0} = \frac{1}{n}\left[ {\sum\limits_{i \in R} {y_{i} } + \sum\limits_{{i \in R^{c} }} {\tilde{y}_{i} } } \right] = \frac{1}{r}\sum\limits_{i = 1}^{r} {y_{i} } = \overline{y}_{r}$$.

It is unbiased for $\overline{Y}$ and its variance for an approximation of order one is,

1 $$V(t_{0} ) = \theta_{r,N} \,\overline{Y}^{2} C_{y}^{2}$$

where, $$\theta_{r,N} = \left( {\frac{1}{r} - \frac{1}{N}} \right)$$$$S_{y}^{2} = \frac{1}{N - 1}\sum\limits_{i = 1}^{N} {(y_{i} } - \overline{Y})^{2}$$, $$C_{y} = \frac{{S_{y} }}{{\overline{Y}}}$$.

The estimator, making the use of auxiliary parameters is the ratio estimator and under the missing observation technique is given by the following imputation method as,

$$y_{.i} = \left\{ \begin{gathered} y_{i} \,\,\,\,\,\,\,{\text{if}}\,\,i \in R \hfill \\ \hfill \\ \hat{\beta }\,\tilde{y}_{i} \,\,\,\,{\text{if }}i \in R^{c} . \hfill \\ \end{gathered} \right.$$

where,$$\hat{\beta }\, = {{\sum\limits_{i = 1}^{r} {y_{i} } } \mathord{\left/ {\vphantom {{\sum\limits_{i = 1}^{r} {y_{i} } } {\sum\limits_{i = 1}^{r} {x_{i} } }}} \right. \kern-0pt} {\sum\limits_{i = 1}^{r} {x_{i} } }}$$.

The resulting estimator of $\overline{Y}$ is presented by,

$$t_{r} = \frac{{\overline{y}_{r} }}{{\overline{x}_{r} }}\overline{x}_{n}$$

where, $$\overline{x}_{r} = \frac{1}{r}\sum\limits_{i \in R} {x_{i} }$$ and $$\overline{x}_{n} = \frac{1}{r}\sum\limits_{i \in S} {x_{i} }$$.

The bias and MSE of $t_{r}$ for an approximation of order one respectively are,

2 $$\begin{gathered} B(t_{r} ) = \theta_{r,n} \overline{Y}\left( {C_{x}^{2} - C_{yx} } \right), \hfill \\ MSE(t_{r} ) = \theta_{r,n} \overline{Y}^{2} \left[ {C_{y}^{2} + C_{x}^{2} - 2C_{yx} } \right] \hfill \\ \end{gathered}$$

where,$$C_{x} = \frac{{S_{x} }}{{\overline{X}}}$$, $$S_{x}^{2} = \frac{1}{N - 1}\sum\limits_{i = 1}^{N} {(x_{i} } - \overline{X})^{2}$$, $$S_{yx} = \frac{1}{N - 1}\sum\limits_{i = 1}^{N} {(y_{i} - \overline{Y})(x_{i} } - \overline{X})$$, $$\rho = \frac{{S_{yx} }}{{S_{y} S_{x} }}$$, $$C_{yx} = \rho C_{y} C_{x}$$, $$\theta_{r,n} = \left( {\frac{1}{r} - \frac{1}{n}} \right)$$, $$\overline{X} = \sum\limits_{i = 1}^{N} {x_{i} }$$.

Singh and Horn^20^ defined the following study variable after utilizing the imputed observation for the responding and non-responding units under consideration as,

$$y_{.i} = \left\{ \begin{gathered} \lambda \frac{n}{r}y_{i} + (1 - \lambda )\hat{\beta }\,x_{i} \,\,\,\,{\text{if}}\,\,i \in R \hfill \\ (1 - \lambda )\hat{\beta }\,x_{i} \,\,\,\,\,\,\,\,\,\,\,\,\,\,\,\,\,\,\,\,\,\,\,\,{\text{if }}i \in R^{c} \,\,. \hfill \\ \end{gathered} \right.$$

Under the imputation method, the resultant estimator of $\overline{Y}$ is as follows,

$$t_{1} = \overline{y}_{r} \left( {\lambda + (1 - \lambda )\frac{{\overline{x}_{n} }}{{\overline{x}_{r} }}} \right)$$

where,$\lambda$ is a characterizing constant to be obtained so that $MSE(t_{1} )$ is least.

The bias and MSE of $t_{1}$ for an approximation of degree one are respectively given by,

$$\begin{gathered} B(t_{1} ) = (1 - \lambda )\theta_{r,n} \overline{Y}\left( {C_{x}^{2} - C_{yx} } \right), \hfill \\ MSE(t_{1} ) = \theta_{r,n} \overline{Y}^{2} C_{y}^{2} + \theta_{r,n} \overline{Y}^{2} \left[ {(1 - \lambda )^{2} C_{x}^{2} - 2(1 - \lambda )C_{yx} } \right] \hfill \\ \end{gathered}$$

The optimum value of $\lambda$ is given by,

$$\lambda_{opt} = 1 - \frac{{C_{yx} }}{{C_{x}^{2} }}.$$

The minimum MSE of $t_{1}$ for $\lambda_{opt}$ is,

3 $$MSE_{\min } (t_{1} ) = \overline{Y}^{2} C_{y}^{2} \left[ {\theta_{r,N} - \theta_{r,n} \rho^{2} } \right].$$

Singh and Deo^21^ utilized the transformed auxiliary information and defined the following variable after using the imputed observation for missing value as,

$$y_{.i} = \left\{ \begin{gathered} y_{i} \,\,\,\,\,\,\,\,\,\,\,\,\,\,\,\,\,\,\,\,\,\,\,\,\,\,\,\,\,\,\,\,\,\,\,\,\,\,\,\,\,\,\,\,\,\,\,\,\,\,\,\,\quad \quad \;\;{\text{if}}\,\,i \in R \hfill \\ \hfill \\ \hfill \\ \overline{y}_{r} \left[ {n\,\left( {\frac{{\overline{x}_{n} }}{{\overline{x}_{r} }}} \right)^{\beta } - r} \right]\frac{{x_{i} }}{{\sum\limits_{{i \in R^{c} }} {x_{i} } }}\,\,\,\,\,\,\,\,\,\,\,{\text{if }}i \in R^{c} . \hfill \\ \end{gathered} \right.$$

The resultant estimator of $\overline{Y}$ is given by,

$$t_{2} = \overline{y}_{r} \,\left( {\frac{{\overline{x}_{n} }}{{\overline{x}_{r} }}} \right)^{\beta } .$$

where, $\beta$ is a characterizing constant and is obtained so that $MSE(t_{1} )$ is least.

The bias and the minimum MSE of $t_{2}$ for an approximation of degree one respectively are,

$$B(t_{2} ) = \theta_{r,n} \overline{Y}\left[ {\frac{\beta (\beta - 1)}{2}C_{x}^{2} - \beta C_{yx} } \right].$$

The optimal value of $\beta$ for which $MSE(t_{2} )$ is least is given by,

$$\beta_{opt} = \rho \frac{{C_{y} }}{{C_{x} }}.$$

The least value of $MSE(t_{2} )$ for $\beta_{opt}$ is,

4 $$MSE_{\min } (t_{2} ) = MSE_{\min } (t_{1} ) - \theta_{r,n} S_{x}^{2} \left( {\frac{{S_{yx} }}{{S_{x}^{2} }} - \frac{{\overline{Y}}}{{\overline{X}}}} \right)^{2} .$$

Kadilar and Cingi^8^ suggested the following regression type estimators of $\overline{Y}$ under the case of missing observations as,

$$t_{3} = \left[ {\overline{y}_{r} + \hat{\beta }(\overline{X} - \overline{x}_{r} )} \right]\frac{{\overline{X}}}{{\overline{x}_{r} }},\;t_{4} = \left[ {\overline{y}_{r} + \hat{\beta }(\overline{X} - \overline{x}_{n} )} \right]\frac{{\overline{X}}}{{\overline{x}_{n} }},\;t_{5} = \left[ {\overline{y}_{r} + \hat{\beta }(\overline{X} - \overline{x}_{n} )} \right]\frac{{\overline{x}_{n} }}{{\overline{x}_{r} }}.$$

The biases and MSEs of $t_{i}$($i = 3,\,4,\,5$) for an approximation of order are respectively given by, $B(t_{3} ) \cong \theta_{n,N} \overline{Y}C_{x}^{2}$, $B(t_{4} ) \cong \theta_{r,N} \overline{Y}C_{x}^{2}$, $B(t_{3} ) \cong \theta_{r,n} \overline{Y}C_{yx}$ and

5 $$MSE_{\min } (t_{3} ) = MSE_{\min } (t_{1} ) - \theta_{r,N} S_{x}^{2} (R^{2} - \beta^{2} ),$$

6 $$MSE_{\min } (t_{4} ) = MSE_{\min } (t_{1} ) - \theta_{n,N} S_{x}^{2} (R^{2} - \beta^{2} ),$$

7 $$MSE_{\min } (t_{5} ) = MSE_{\min } (t_{1} ) - \theta_{r,n} [(R + \beta )^{2} S_{x}^{2} - 2(R + \beta )S_{yx} ],$$

where, $$R = \frac{{\overline{Y}}}{{\overline{X}}}$$ and $$\beta = \frac{{S_{yx} }}{{S_{x}^{2} }}$$.

Singh^22^ suggested a new modified imputation method for estimation of $\overline{Y}$ and suggested the following variable as,

$$y_{.i} = \left\{ \begin{gathered} \,y_{i} \,\,\,\,\,\,\,\,\,\,\,\,\,\,\,\,\,\,\,\,\,\,\,\,\,\,\,\,\,\,\,\,\,\,\,\,\,\,\,\,\,\,\,\,\,\,\,\,\,\,\,\,\quad \quad \quad \quad \quad \quad \;{\text{if}}\,\,i \in R \hfill \\ \overline{y}_{r} \left[ {\frac{{(n - r)\overline{x}_{n} + \alpha \,r(\overline{x}_{n} - \overline{x}_{r} )}}{{\alpha \,\overline{x}_{n} - (1 - \alpha )\overline{x}_{r} }}} \right]\frac{{x_{i} }}{{\sum\limits_{{i \in R^{c} }} {x_{i} } }}\,\,\,\,{\text{if }}i \in R^{c} \hfill \\ \end{gathered} \right..$$

The resultant estimator of $\overline{Y}$ is given by,

$$t_{6} = \frac{{\overline{y}_{r} \overline{x}_{n} \,}}{{\alpha \,\overline{x}_{r} + (1 - \alpha )\overline{x}_{n} }}$$

where, $\alpha$ is a scalar to be obtained so that $MSE(t_{6} )$ is least.

The bias and MSE of $t_{6}$ for an approximation of degree one respectively are,

$$\begin{gathered} B(t_{6} ) = \overline{Y}\left[ {\theta_{n,N} C_{yx} + \alpha^{2} \theta_{r,n} C_{x}^{2} + (1 - \alpha )^{2} \theta_{n,N} C_{x}^{2} - \alpha (\theta_{r,n} C_{yx} + \theta_{n,N} C_{x}^{2} )} \right. \hfill \\ \left. {\quad \quad \quad \quad + 2\alpha (\alpha - 1)\theta_{n,N} C_{x}^{2} - (1 - \alpha )\theta_{n,N} (C_{yx} + C_{x}^{2} )} \right] \hfill \\ \end{gathered}$$

The optimal value of $\alpha$ for which $MSE(t_{6} )$ is least is given by $\alpha_{opt} = \rho {{C_{y} } \mathord{\left/ {\vphantom {{C_{y} } {C_{x} }}} \right. \kern-0pt} {C_{x} }}$.

The least value of $MSE(t_{6} )$ for $\alpha_{opt}$ is,

8 $$MSE_{\min } (t_{6} ) = MSE_{\min } (t_{1} ) - \theta_{r,n} S_{x}^{2} \left( {\frac{{S_{yx} }}{{S_{x}^{2} }} - \frac{{\overline{Y}}}{{\overline{X}}}} \right)^{2} .$$

Singh et al.^23^ suggested a new imputation method using exponential function as,

$$y_{.i} = \left\{ \begin{gathered} \,\alpha \frac{n}{r}y_{i} + (1 - \alpha )\overline{y}_{r} \exp \left( {\frac{{\overline{X} - \overline{x}_{r} }}{{\overline{X} + \overline{x}_{r} }}} \right)\,\,\,\,\,\,{\text{if}}\,\,i \in R \hfill \\ (1 - \alpha )\overline{y}_{r} \exp \left( {\frac{{\overline{X} - \overline{x}_{r} }}{{\overline{X} + \overline{x}_{r} }}} \right)\,\,\,\,\,\,\,\,\,\,\,\,\,\,\,\,\,\,\,\,\,\,\,\,\,\,\,{\text{if }}i \in R^{c} . \hfill \\ \end{gathered} \right.$$

The resultant estimator of $\overline{Y}$ under imputation method is given by,

$$t_{7} = \alpha \,\overline{y}_{r} + (1 - \alpha )\overline{y}_{r} \exp \left( {\frac{{\overline{X} - \overline{x}_{r} }}{{\overline{X} + \overline{x}_{r} }}} \right).$$

where, $\alpha$ is a constant to be obtained so that $MSE(t_{7} )$ is least.

The bias and MSE of $t_{7}$ for an approximation of degree one respectively are,

$$\begin{gathered} B(t_{7} ) = (1 - \alpha )\theta_{r,N} \overline{Y}\left( {\frac{3}{8}C_{x}^{2} - \frac{1}{2}C_{yx} } \right), \hfill \\ MSE(t_{7} ) = \theta_{r,N} \overline{Y}^{2} \left[ {C_{y}^{2} + \frac{{(1 - \alpha )^{2} }}{4}C_{x}^{2} - (1 - \alpha )C_{yx} ]} \right]. \hfill \\ \end{gathered}$$

The optimal value of $\alpha$ is given by,

$$\alpha_{opt} = 1 - 2\frac{{C_{yx} }}{{C_{x}^{2} }}.$$

The least MSE of $t_{7}$ for $\alpha_{opt}$ is given by,

9 $$MSE_{\min } (t_{7} ) = MSE_{\min } (t_{1} ) - \theta_{r,n} S_{x}^{2} \left( {\frac{{S_{yx} }}{{S_{x}^{2} }} - \frac{{\overline{Y}}}{{\overline{X}}}} \right)^{2} .$$

Gira^10^ suggested a novel imputation method for the estimation of $\overline{Y}$ as,

$$y_{.i} = \left\{ \begin{gathered} \,y_{i} \,\,\,\,\,\,\,\,\,\,\,\,\,\,\,\,\,\,\,\,\,\,\,\,\,\,\,\,\,\,\,\,\,\,\,\,\,\,\,\,\,\,\,\,\,\,\,\,\,\,\,\,\,\quad \quad \;{\text{if}}\,\,i \in R \hfill \\ \overline{y}_{r} \left[ {n\frac{{(\delta - \overline{x}_{r} )}}{{(\delta - \overline{x}_{n} )}} - r} \right]\frac{{x_{i} }}{{\sum\limits_{{i \in R^{c} }} {x_{i} } }}\,\,\,\,\,\,\,\,{\text{if }}i \in R^{c} . \hfill \\ \end{gathered} \right.$$

The resultant estimator of $\overline{Y}$ is given by,

$$t_{8} = \overline{y}_{r} \left[ {\frac{{(\delta - \overline{x}_{r} )}}{{(\delta - \overline{x}_{n} )}}} \right].$$

where, $\delta$ is the scalar to be obtained so that $MSE(t_{8} )$ is least.

The bias of $t_{8}$ is given by,

$$B(t_{8} ) = - \theta_{r,n} \frac{{\overline{X}\,\overline{Y}}}{{\delta - \overline{X}}}C_{yx} .$$

The optimal value of $\delta$ for which $MSE(t_{8} )$ is least, is given by,

$$\delta_{opt} = \overline{X}\left( {\frac{{C_{x} }}{{\rho C_{y} }} - 1} \right).$$

The least value of $MSE(t_{8} )$ for $\delta_{opt}$ is,

10 $$MSE_{\min } (t_{8} ) = V(t_{0} ) - \theta_{r,n} \overline{Y}^{2} \rho^{2} C_{y}^{2} .$$

Singh et al.^4^ worked on a new imputation method as,

$$y_{.i} = \left\{ \begin{gathered} y_{i} \,\,\,\,\,\,\,\,\,\,\,\,\,\,\,\,\,\,\,\,\,\,\,\,\,\,\,\,\,\,\,\,\,\,\,\,\,\,\,\,\,\,\,\,\,\,\,\,\,\,\,\,\,\,\,\,\,\,\,\,\,\,\,\,\,\,\,\,\,\,\,\,\,\,\,\,\,\,\,\,\,\,\,\,\,\,\,\,\,\,\,\,\,\,\,\,\,\,\,\,\,\,\,\,\,\,\,\,\,\quad \quad \quad \quad \quad \quad {\text{if}}\,\,i \in R \hfill \\ \overline{y}_{r} \left[ {\frac{{\{ m\,(n + r) - r\} \overline{x}_{n} + \{ (1 - m)n - mr\} \overline{x}_{n} }}{{m\,\overline{x}_{n} - (1 - m)\overline{x}_{r} }}} \right]\frac{{x_{i} }}{{\sum\limits_{{i \in R^{c} }} {x_{i} } }}\,\,\,\,\,\,\,\,\,\,\,\,\,\,\,\,{\text{if }}i \in R^{c} . \hfill \\ \end{gathered} \right.$$

The resultant point estimator of $\overline{Y}$ is given by,

$$t_{9} = \overline{y}_{r} \left[ {\frac{{m\overline{x}_{r} + (1 - m)\overline{x}_{n} }}{{m\overline{x}_{n} + (1 - m)\overline{x}_{r} }}} \right].$$

The bias and MSE of $t_{9}$ for an approximation of degree one respectively are,

$$\begin{gathered} B(t_{9} ) = \theta_{r,n} \overline{Y}\left[ {(2m^{2} - 3m + 1)C_{x}^{2} + (2m - 1)C_{yx} } \right], \hfill \\ MSE(t_{9} ) = \overline{Y}^{2} \left[ {\theta_{r,N} C_{y}^{2} + (1 - 2m)^{2} \theta_{r,n} C_{x}^{2} - 2(1 - 2m)\theta_{r,n} C_{yx} } \right]. \hfill \\ \end{gathered}$$

The optimum value of $m$ is given by,

$$m_{opt} = \frac{1}{2}\left( {1 - \rho \frac{{C_{y} }}{{C_{x} }}} \right).$$

The least value of $MSE(t_{9} )$ for $m_{opt}$ is,

11 $$MSE_{\min } (t_{9} ) = V(t_{0} ) - \theta_{r,n} \overline{Y}^{2} \rho^{2} C_{y}^{2} .$$

Aliyu et al.^24^ suggested a new imputation method for the estimation of $\overline{Y}$ as,

$$y_{.i} = \left\{ \begin{gathered} y_{i} \,\,\,\,\,\,\,\,\,\,\,\,\,\,\,\,\,\,\,\,\,\,\,\,\,\,\,\,\,\,\,\,\,\,\,\,\,\,\,\,\,\,\,\,\,\,\,\,\,\,\,\,\,\,\,\,\,\,\,\,\,\,\,\,\,\,\,\,\,\,\,\,\,\,\,\,\,\,\,\,\,\,\,\,\,\,\,\,\,\,\,\,\,\,\,\,\,\,\,\,\,\,\,\,\,\,\,\,\,\,\,\,\,\,\,\,\,\quad \quad \quad \quad \quad \;{\text{if}}\,\,i \in R \hfill \\ \,\frac{1}{n - r}\left[ {n\left\{ {\alpha \,\overline{y}_{r} + (1 - \alpha )\overline{y}_{r} \frac{{\overline{x}_{r} }}{{\overline{X}}}} \right\}\exp \left( {\frac{{\overline{X} - \overline{x}_{r} }}{{\overline{X} + \overline{x}_{r} }}} \right) - r\overline{y}_{r} } \right]\,\,\,\,\,\,\,\,\,\,\,\,\,\,\,\,\,{\text{if }}i \in R^{c} . \hfill \\ \end{gathered} \right.$$

The resultant estimator of $\overline{Y}$ is,

$$t_{10} = \left\{ {\alpha \,\overline{y}_{r} + (1 - \alpha )\overline{y}_{r} \frac{{\overline{x}_{r} }}{{\overline{X}}}} \right\}\exp \left( {\frac{{\overline{X} - \overline{x}_{r} }}{{\overline{X} + \overline{x}_{r} }}} \right).$$

The bias and MSE of $t_{10}$ for an approximation of degree one respectively are,

$$\begin{gathered} B(t_{10} ) = \theta_{r,N} \overline{Y}\left[ { - \left( {\frac{1}{8} - \alpha } \right)C_{x}^{2} + \left( {\frac{1}{2} + \alpha } \right)C_{yx} } \right]. \hfill \\ MSE(t_{9} ) = \theta_{r,N} \overline{Y}^{2} \left[ {C_{y}^{2} + \left( {\frac{1}{2} - \alpha } \right)^{2} C_{x}^{2} + 2\left( {\frac{1}{2} - \alpha } \right)C_{yx} } \right]. \hfill \\ \end{gathered}$$

The optimum value of $\alpha$ is given by,

$$\alpha_{opt} = \frac{1}{2} + \rho \frac{{C_{y} }}{{C_{x} }}$$

The least value of $MSE(t_{10} )$ for $\alpha_{opt}$ is,

12 $$MSE_{\min } (t_{10} ) = \theta_{r,N} \overline{Y}^{2} C_{y}^{2} (1 - \rho^{2} ).$$

## Suggested computational strategy

Searls^25^ suggested and proved that an estimator which is a constant multiple of sample mean estimator, is more efficient than the sample mean estimator. This has also been proven by various authors for ratio and product estimators. Therefore, motivated by Searls^25^ and Aliyu et al.^24^, we suggest the following imputation strategy for the estimation of $\overline{Y}$ as,

$$y_{.i} = \left\{ \begin{gathered} y_{i} \,\,\,\,\,\,\,\,\,\,\,\,\,\,\,\,\,\,\,\,\,\,\,\,\,\,\,\,\,\,\,\,\,\,\,\,\,\,\,\,\,\,\,\,\,\,\,\,\,\,\,\,\,\,\,\,\,\,\,\,\,\,\,\,\,\,\,\,\,\,\,\,\,\,\,\,\,\,\,\,\,\,\,\,\,\,\,\,\,\,\,\,\,\,\,\,\,\,\,\,\,\,\,\,\,\,\,\,\,\,\quad \quad \quad \quad \;\;{\text{if}}\,\,i \in R \hfill \\ \,\frac{1}{n - r}\left[ {n\left\{ {\kappa_{1} \,\overline{y}_{r} + \kappa_{2} \,\overline{y}_{r} \frac{{\overline{x}_{r} }}{{\overline{X}}}} \right\}\exp \left( {\frac{{\overline{X} - \overline{x}_{r} }}{{\overline{X} + \overline{x}_{r} }}} \right) - r\overline{y}_{r} } \right]\,\,\,\,\,\,\,\,\,\,\,\,\,\,\,\,\,{\text{if }}i \in R^{c} . \hfill \\ \end{gathered} \right.$$

The resultant point estimator of $\overline{Y}$ is,

$$t_{p} = \left\{ {\kappa_{1} \,\,\overline{y}_{r} + \kappa_{2} \,\overline{y}_{r} \frac{{\overline{x}_{r} }}{{\overline{X}}}} \right\}\exp \left( {\frac{{\overline{X} - \overline{x}_{r} }}{{\overline{X} + \overline{x}_{r} }}} \right)$$

where, $\kappa_{1} \,$ and $\kappa_{2} \,$ are the Searls constants to be obtained so that the MSE of $t_{p}$ is least and $\kappa_{1} + \kappa_{2} \ne 1$. If $\kappa_{1} + \kappa_{2} = 1$, then the suggested estimator reduced to Aliyu et al.^24^ estimator.

The properties of the sampling distribution of $t_{p}$, the standard approximations are given as,

$$\overline{y}_{r} \, = \overline{Y}(1 + e_{0} )$$, $$\overline{x}_{r} \, = \overline{X}(1 + e_{1} )$$.

such that $E(e_{0} ) = E(e_{1} ) = 0$ and $E(e_{0}^{2} ) = \theta_{r,N} C_{y}^{2}$, $E(e_{1}^{2} ) = \theta_{r,N} C_{x}^{2}$, $E(e_{0} e_{1} ) = \theta_{r,N} C_{yx}$.

Expressing $t_{p}$ in terms of $e_{i} ^{\prime}s$($i = 0,\,1$), expanding and retaining the terms for an approximation of order one, we have,

$$\begin{gathered} t_{p} = \left[ {\kappa_{1} \overline{Y}(1 + e_{0} ) + \kappa_{2} \overline{Y}(1 + e_{0} )\frac{{\overline{X}(1 + e_{1} )}}{{\overline{X}}}} \right]\exp \left[ {\frac{{\overline{X} - \overline{X}(1 + e_{1} )}}{{\overline{X} + \overline{X}(1 + e_{1} )}}} \right], \hfill \\ \quad = [\kappa_{1} \overline{Y}(1 + e_{0} ) + \kappa_{2} \overline{Y}(1 + e_{0} )(1 + e_{1} )]\exp \left[ {\frac{{ - e_{1} }}{{2 + e_{1} }}} \right], \hfill \\ \quad = \overline{Y}[\kappa_{1} (1 + e_{0} ) + \kappa_{2} (1 + e_{0} )(1 + e_{1} )]\exp \left[ { - \frac{{e_{1} }}{2} + \frac{{e_{1}^{2} }}{4}} \right], \hfill \\ \quad = \overline{Y}[\kappa_{1} (1 + e_{0} ) + \kappa_{2} (1 + e_{0} + e_{1} + e_{0} e_{1} )]\left[ {1 - \frac{{e_{1} }}{2} + \frac{{3e_{1}^{2} }}{8}} \right], \hfill \\ \quad = \left[ {\kappa_{1} \left( {1 + e_{0} - \frac{{e_{1} }}{2} - \frac{{e_{0} e_{1} }}{2} + \frac{{3e_{1}^{2} }}{8}} \right) + \kappa_{2} \left( {1 + e_{0} + \frac{{e_{1} }}{2} + \frac{{e_{0} e_{1} }}{2} - \frac{{e_{1}^{2} }}{8}} \right)} \right]. \hfill \\ \end{gathered}$$

Subtracting $\overline{Y}$ on both sides of above equation, we get,

13 $$t_{p} - \overline{Y} = \overline{Y}\left[ {\kappa_{1} \left( {1 + e_{0} - \frac{{e_{1} }}{2} - \frac{{e_{0} e_{1} }}{2} + \frac{{3e_{1}^{2} }}{8}} \right) + \kappa_{2} \left( {1 + e_{0} + \frac{{e_{1} }}{2} + \frac{{e_{0} e_{1} }}{2} - \frac{{e_{1}^{2} }}{8}} \right) - 1} \right].$$

Taking expectation on both sides and putting values of different expectations, we have bias of $t_{p}$ as,

$$B(t_{p} ) = \overline{Y}\left[ {\kappa_{1} \left( {1 - \frac{1}{2}\theta_{r,N} C_{yx} + \frac{3}{8}\theta_{r,N} C_{x}^{2} } \right) + \kappa_{2} \left( {1 + \frac{1}{2}\theta_{r,N} C_{yx} - \frac{1}{8}\theta_{r,N} C_{x}^{2} } \right) - 1} \right].$$

Squaring on both sides of (13), simplifying and putting the terms for an approximation of degree one, we get the MSE of $t_{p}$ as,

$$\begin{gathered} MSE(t_{p} ) = \overline{Y}^{2} E\left[ {\kappa_{1} \left( {1 + e_{0} - \frac{{e_{1} }}{2} - \frac{{e_{0} e_{1} }}{2} + \frac{{3e_{1}^{2} }}{8}} \right) + \kappa_{2} \left( {1 + e_{0} + \frac{{e_{1} }}{2} + \frac{{e_{0} e_{1} }}{2} - \frac{{e_{1}^{2} }}{8}} \right) - 1} \right]^{2} , \hfill \\ \quad \quad \quad \quad \;\; = \overline{Y}^{2} E\left[ {1 + \kappa_{1}^{2} \left( {1 + e_{0}^{2} + e_{1}^{2} - 2e_{0} e_{1} } \right) + \kappa_{2}^{2} \left( {1 + e_{0}^{2} + 2e_{0} e_{1} } \right) + 2\kappa_{1} \kappa_{2} \left( {1 + e_{0}^{2} } \right)} \right. \hfill \\ \left. {\quad \quad \quad \quad \quad \; - 2\kappa_{1} \left( {1 - \frac{1}{2}e_{0} e_{1} + \frac{3}{8}e_{1}^{2} } \right) - 2\kappa_{2} \left( {1 - \frac{1}{2}e_{0} e_{1} - \frac{1}{8}e_{1}^{2} } \right)} \right]. \hfill \\ \end{gathered}$$

Putting the values of different expectations, we get,

14 $$\begin{gathered} MSE(t_{p} ) = \overline{Y}^{2} \left[ {1 + \kappa_{1}^{2} \left\{ {1 + \theta_{r,N} \left( {C_{y}^{2} + C_{x}^{2} - 2C_{yx} } \right)} \right\} + \kappa_{2}^{2} \left\{ {1 + \theta_{r,N} \left( {C_{y}^{2} - 2C_{yx} } \right)} \right\}2\kappa_{1} \kappa_{2} \left( {1 + \theta_{r,N} C_{y}^{2} } \right)} \right. \hfill \\ \left. {\quad \quad \quad \quad \quad - 2\kappa_{1} \left( {1 - \frac{1}{2}\theta_{r,N} C_{yx} + \frac{3}{8}\theta_{r,N} C_{x}^{2} } \right) - 2\kappa_{2} \left( {1 - \frac{1}{2}\theta_{r,N} C_{yx} - \frac{1}{8}\theta_{r,N} C_{x}^{2} } \right)} \right], \hfill \\ MSE(t_{p} ) = \overline{Y}^{2} \left[ {1 + \kappa_{1}^{2} A + \kappa_{2}^{2} B + 2\kappa_{1} \kappa_{2} C - 2\kappa_{1} D - 2\kappa_{2} F} \right] \hfill \\ \end{gathered}$$

where $$A = \{ 1 + \theta_{r,N} (C_{y}^{2} + C_{x}^{2} - 2C_{yx} )\}$$, $$B = \{ 1 + \theta_{r,N} (C_{y}^{2} - 2C_{yx} )\}$$, $$C = (1 + \theta_{r,N} C_{y}^{2} )$$, $$D = \left( {1 - \frac{1}{2}\theta_{r,N} C_{yx} + \frac{3}{8}\theta_{r,N} C_{x}^{2} } \right)$$, $$F = \left( {1 - \frac{1}{2}\theta_{r,N} C_{yx} - \frac{1}{8}\theta_{r,N} C_{x}^{2} } \right)$$.

The optimal values of $\kappa_{1} \,$ and $\kappa_{2}$, which reduces the MSE of $t_{p}$ respectively are,

$$\kappa_{1(opt)} \, = \frac{BD - CF}{{AB - C^{2} }}\;{\text{and}}\;\kappa_{2(opt)} = \frac{AF - DC}{{AB - C^{2} }}.$$

The minimum value of $MSE(t_{p} )$ for the optimum values of $\kappa_{1} \,$ and $\kappa_{2}$ is,

15 $$\begin{gathered} MSE_{\min } (t_{p} ) = \overline{Y}^{2} \left[ {1 - \frac{{\left\{ \begin{gathered} 2(AF - DC)F + 2(BD - CF)D - 2(AF - DC)(BD - CF)C \hfill \\ - (BD - CF)^{2} A - (AF - DC)^{2} B \hfill \\ \end{gathered} \right\}}}{{(AB - C^{2} )^{2} }}} \right], \hfill \\ MSE_{\min } (t_{p} ) = \overline{Y}^{2} \left[ {1 - \frac{L}{{M^{2} }}} \right] \hfill \\ \end{gathered}$$

where,

$$\begin{gathered} L = \left\{ \begin{gathered} 2(AF - DC)F + 2(BD - CF)D - 2(AF - DC)(BD - CF)C \hfill \\ - (BD - CF)^{2} A - (AF - DC)^{2} B \hfill \\ \end{gathered} \right\}, \hfill \\ M = (AB - C^{2} ). \hfill \\ \end{gathered}$$

## Theoretical efficiency comparison

In this section, the introduced estimator has been compared with the estimators of $\overline{Y}$ in competition under imputation methods in terms of their efficiencies. The efficiency conditions for which the introduced estimator is more efficient than the completing estimators are obtained.

The introduced estimator $t_{p}$ is better than $t_{0}$ under imputation method if,

16 $$\begin{gathered} V(t_{0} ) - MSE_{\min } (t_{p} ) > 0,\;{\text{or}} \hfill \\ \theta_{r,N} \,C_{y}^{2} - \left[ {1 - \frac{L}{{M^{2} }}} \right] > 0. \hfill \\ \end{gathered}$$

The introduced estimator $t_{p}$ has lesser MSE than the usual ratio estimator $t_{r}$ for the condition if,

17 $$\begin{gathered} MSE(t_{r} ) - MSE_{\min } (t_{p} ) > 0,\;{\text{or}} \hfill \\ \theta_{r,n} [C_{y}^{2} + C_{x}^{2} - 2C_{yx} ] + \frac{L}{{M^{2} }} > 1. \hfill \\ \end{gathered}$$

The suggested estimator $t_{p}$ performs better than the Singh and Horn^20^ estimator $t_{1}$ for the condition if,

18 $$\begin{gathered} MSE_{\min } (t_{1} ) - MSE_{\min } (t_{p} ) > 0,\;{\text{or}} \hfill \\ C_{y}^{2} [\theta_{r,N} - \theta_{r,n} \rho^{2} ] + \frac{L}{{M^{2} }} > 1. \hfill \\ \end{gathered}$$

The proposed estimator $t_{p}$ is more efficient than the Singh and Deo^21^ estimator $t_{2}$ if,

19 $$\begin{gathered} MSE_{\min } (t_{2} ) - MSE_{\min } (t_{p} ) > 0,\;{\text{or}} \hfill \\ MSE_{\min } (t_{1} ) - \theta_{r,n} \,S_{x}^{2} \left( {\frac{{S_{yx} }}{{S_{x}^{2} }} - \frac{{\overline{Y}}}{{\overline{X}}}} \right)^{2} + \frac{L}{{M^{2} }} > 1. \hfill \\ \end{gathered}$$

The introduced estimator $t_{p}$ has lesser MSE than the Kadilar and Cingi^8^ estimators $t_{i} \,\,;\,\,(i = 3,\,4,\,5)$) under the conditions if,

$$MSE_{\min } (t_{i} ) - MSE_{\min } (t_{p} ) > 0;\;i = 3,4,5;\;{\text{or}}$$

20 $$MSE_{\min } (t_{1} ) - \theta_{r,N} S_{x}^{2} (R^{2} - \beta^{2} ) + \frac{L}{{M^{2} }} > 1,$$

21 $$MSE_{\min } (t_{1} ) - \theta_{n,N} S_{x}^{2} (R^{2} - \beta^{2} ) + \frac{L}{{M^{2} }} > 1,$$

22 $$MSE_{\min } (t_{1} ) - \theta_{r,n} \left[ {(R + \beta )^{2} S_{x}^{2} - 2(R + \beta )S_{yx} } \right] + \frac{L}{{M^{2} }} > 1.$$

The suggested estimator $t_{p}$ performs better than the Singh^22^ estimator $t_{6}$ for the condition if,

23 $$\begin{gathered} MSE_{\min } (t_{6} ) - MSE_{\min } (t_{p} ) > 0,\;{\text{or}} \hfill \\ MSE_{\min } (t_{1} ) - \theta_{r,n} S_{x}^{2} \left( {\frac{{S_{yx} }}{{S_{x}^{2} }} - \frac{{\overline{Y}}}{{\overline{X}}}} \right)^{2} + \frac{L}{{M^{2} }} > 1. \hfill \\ \end{gathered}$$

The proposed estimator $t_{p}$ is more efficient than the Singh et al.^23^ estimator $t_{7}$ for the condition if,

24 $$\begin{gathered} MSE_{\min } (t_{7} ) - MSE_{\min } (t_{p} ) > 0,\;{\text{or}} \hfill \\ MSE_{\min } (t_{1} ) - \theta_{r,n} S_{x}^{2} \left( {\frac{{S_{yx} }}{{S_{x}^{2} }} - \frac{{\overline{Y}}}{{\overline{X}}}} \right)^{2} + \frac{L}{{M^{2} }} > 1. \hfill \\ \end{gathered}$$

The introduced estimator $t_{p}$ performs better than the Gira^10^ estimator $t_{8}$ for the condition if,

25 $$\begin{gathered} MSE_{\min } (t_{8} ) - MSE_{\min } (t_{p} ) > 0,\;{\text{or}} \hfill \\ V(t_{0} ) - \theta_{r,n} \overline{Y}^{2} \rho^{2} C_{y}^{2} + \frac{L}{{M^{2} }} > 1. \hfill \\ \end{gathered}$$

The suggested estimator $t_{p}$ has lesser MSE than the Singh et al.^4^ estimator $t_{9}$ if the following condition is satisfied.

26 $$\begin{gathered} MSE_{\min } (t_{9} ) - MSE_{\min } (t_{p} ) > 0,\;{\text{or}} \hfill \\ V(t_{0} ) - \theta_{r,n} \overline{Y}^{2} \rho^{2} C_{y}^{2} + \frac{L}{{M^{2} }} > 1. \hfill \\ \end{gathered}$$

The introduced estimator $t_{p}$ is more efficient than the Aliyu et al.^24^ estimator $t_{10}$ under the condition if,

27 $$\begin{gathered} MSE_{\min } (t_{10} ) - MSE_{\min } (t_{p} ) > 0,\;{\text{or}} \hfill \\ \theta_{r,N} \overline{Y}^{2} C_{y}^{2} (1 - \rho^{2} ) + \frac{L}{{M^{2} }} > 1. \hfill \\ \end{gathered}$$

## Empirical study

To verify the efficiency conditions of the introduced estimator over the estimators in competition, we have considered the following three natural populations. The data of Population-1 has been taken from Murthy^26^, while Population-2 has been taken into account from Cochran^27^ and the data set of Population-3 has been obtained from Sarndal et al.^28^ for both study and the auxiliary variables. These populations are of different natures as per their populations sizes and, we have taken samples of different sizes to see the performances of the competing and introduced estimators. The parameters of the considered three populations along with their sources are presented in Table 1.

**Table 1:** Parameters of the three natural populations.

Population-1: Murthy^26^	Population-2: Cochran^27^	Population-3: Sarndal et al.^28^
$N = 80$, $n = 25$,$r = 20$	$N = 10$, $n = 5$,$r = 4$	$N = 284$, $n = 35$,$r = 25$
$\overline{Y} = 5182.638$, $\overline{X} = 285.125$	$\overline{Y} = 56.900$, $\overline{X} = 54.296$	$\overline{Y} = 29.360$, $\overline{X} = 245.088$
$C_{y} = 0.354$, $C_{x} = 0.949$	$C_{y} = 0.184$, $C_{x} = 0.162$	$C_{y} = 1.760$, $C_{x} = 2.430$
$\beta_{1} (x) = 0.949$	$\beta_{1} (x) = 0.496$	$\beta_{1} (x) = 8.770$
$\beta_{2} (x) = 3.536$, $\rho_{yx} = 0.914$	$\beta_{2} (x) = 2.593$, $\rho_{yx} = 0.924$	$\beta_{2} (x) = 88.880$, $\rho_{yx} = 0.961$

Table 2 represents the MSE of different estimators in competition and the introduced estimator along with the Percentage Relative Efficiency (PRE) of various estimators with respect to $t_{0}$ for all the three natural populations.

**Figure 1:**
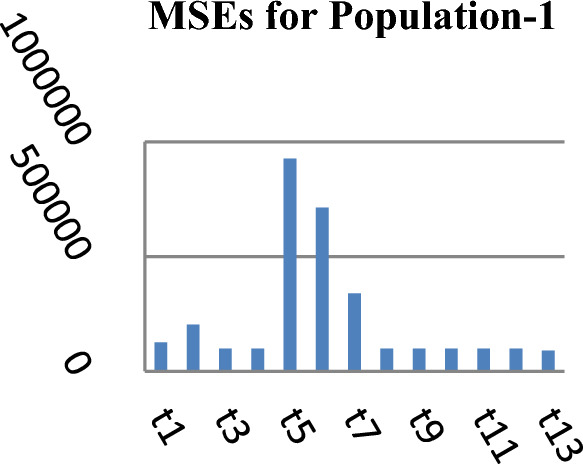
MSEs of Different estimators.

**Figure 2:**
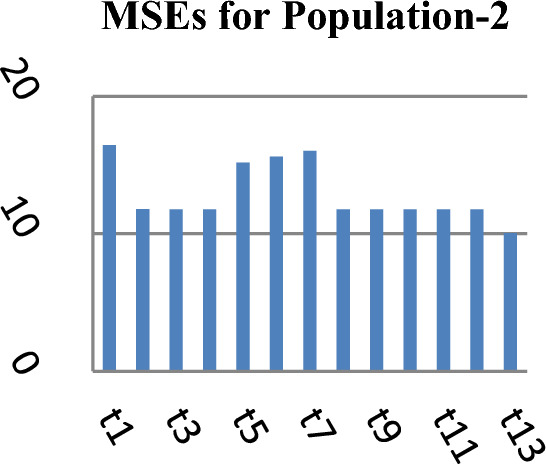
MSEs of Different estimators.

**Figure 3:**
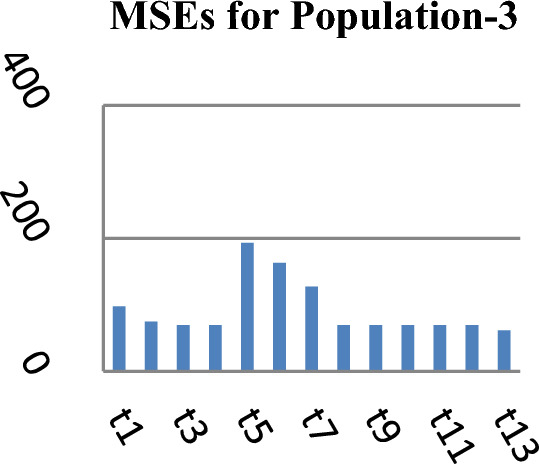
MSEs of Different estimators.

The following Figs. 1, 2 and 3 represent the MSEs of the estimators in competition and the introduced estimators for the three real natural populations respectively.

**Figure 4:**
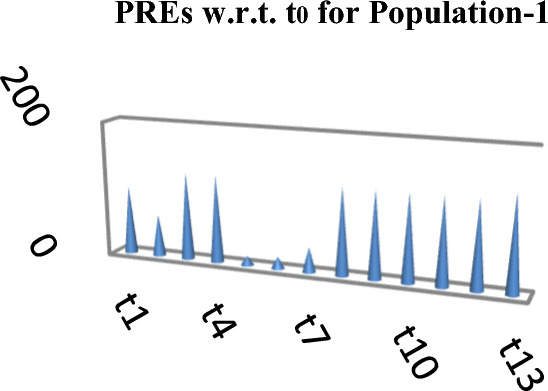
PREs of Different estimators.

**Figure 5:**
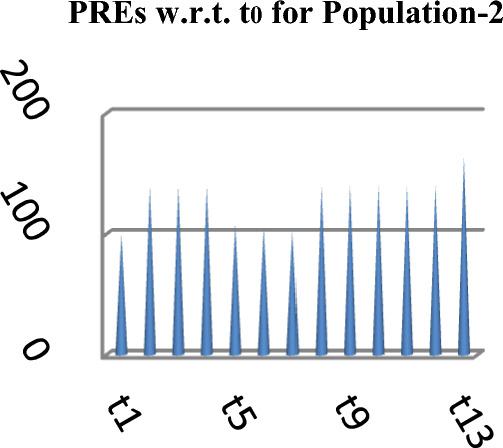
PREs of Different estimators.

**Figure 6:**
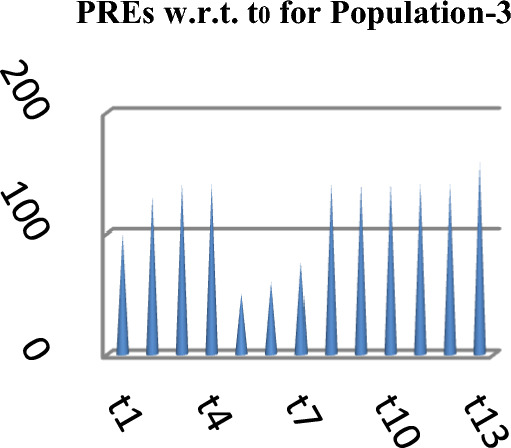
PREs of Different estimators.

The following Figs. 4, 5 and 6 represent the PREs of the competing and the suggested estimators with respect to $t_{0}$ for the three natural populations respectively.

## Simulation study

In this section, an artificial data has been generated for the comparison of competing and introduced imputation methods for the large simulated population to see the nature of different estimators under comparison. We have generated a population using the same parameters of the real population-3. We have taken a sizable sample to examine the nature of the suggested estimator and other estimators for large samples, rather than taking into account the changing sample size in the simulation study because it is well-established in the literature that the estimate becomes closer to the true parameter as the sample size increases. We created the population using a normal distribution because it is widely known that all theoretical and sampling distributions approach a normal distribution for big sample sizes. This means that the findings of more complicated real-world scenarios with various sample sizes would remain unchanged. A bivariate normal distribution with mean vectors and a variance–covariance matrix is used to construct the population as:

Means of [$Y,\,X$] as $\mu = [29.360,\,245.088]$.

Variances and covariance of $[Y,\,X]$ as $$\sigma^{2} = \left[ \begin{gathered} \,\,2670.161\,\,\,\,\,\,29574.704 \hfill \\ 29574.704\,\,\,354696.288 \hfill \\ \end{gathered} \right]$$.

Correlation $$\rho_{yx} = 0.961$$.

The following steps have been used for the simulation of the required population:

- A bivariate normal distribution of X and Y of size $N = 5000$ have been generated through these parameters using R Program.
- The parameters have been computed for this simulated population of size $N = 5000$.
- A sample of size $n = 200$ has been selected from this simulated population with response rate $r = 160$.
- Sample statistics that is sample mean, sample variance and the values of the introduced and competing estimators $t_{i}$, $i = 0,\,1,\,...,\,10,\,p$ of $\overline{Y}$ are calculated for this sample under imputation technique.
- Steps (c) and (d) are repeated $m = 50,000$ times.
- The MSE of every estimator $t_{i}$ is calculated through the formula, $MSE(t_{i} ) = \frac{1}{m}\sum\limits_{j = 1}^{m} {(t_{ij} - \overline{Y})^{2} }$.
- The PRE of each of the estimator $t_{i}$ with respect to $t_{0}$ has been calculated using the formula:
- $$PRE(t_{i} ) = \frac{{MSE(t_{0} )}}{{MSE(t_{i} )}} \times 100$$, $$i = 1,2,...,10,p$$

Table 3 represents the PRE of various estimators of under imputation methods with respect to for the simulated population.

**Figure 7:**
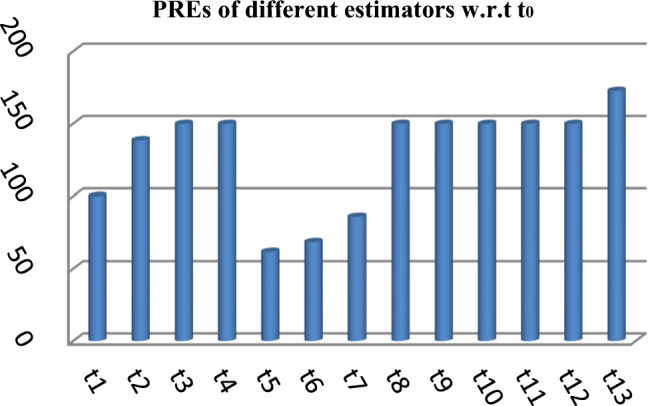
PREs of different estimators for the simulated population.

These results are also prsesnted in the form of graph in Fig. 7 given below as,

## Results and discussion

From Table 2, it may be observed that the MSEs of the competing estimator of $\overline{Y}$ under imputation methods lie in the intervals [98215.14, 926,966.20], [11.77, 16.44] and [69.22, 97.40] while for the suggested estimator, these are 90,056.57, 10.04 and 61.75 for Population-1, Population-2 and Population-3 respectively. On the other hand the PREs of various estimators with respect to $t_{0}$ lie in the intervals [13.63, 128.66], [102.62, 139.68] and [50.43, 140.41] while the PREs of the suggested estimator are 140.32, 163.75 and 158.41 for Population-1, Population-2 and Population-3 respectively. The same results may also be verified from the figures from Figs. 1, 2, 3, 4, 5, and 6 for the three real populations under consideration. It may also be observed from Table 3 that the PREs of various estimators in competition with respect to $t_{0}$ lie in the interval [61.76, 149.81] and for the introduced estimator is 172.68, which may also be verified from Fig. 7 for the simulated data.

**Table 2:** MSE and PRE of different estimators with respect to $t_{0}$.

Estimator	Population-1		Population-2		Population-3	
	MSE	PRE	MSE	PRE	MSE	PRE
$t_{0}$	126,366.00	100.00	16.44	100.00	97.40	100.00
$t_{r}$	203,055.80	62.23	11.78	139.56	74.60	130.56
$t_{1}$	98,215.14	128.66	11.77	139.68	69.22	140.71
$t_{2}$	98,215.14	128.66	11.77	139.68	69.22	140.71
$t_{3}$	926,966.20	13.63	15.17	108.37	193.13	50.43
$t_{4}$	713,472.80	17.71	15.60	105.38	163.14	59.71
$t_{5}$	339,859.40	37.18	16.02	102.62	127.39	76.46
$t_{6}$	98,215.14	128.66	11.77	139.68	69.22	140.71
$t_{7}$	98,215.14	128.66	11.77	139.68	69.22	140.71
$t_{8}$	98,215.14	128.66	11.77	139.68	69.22	140.71
$t_{9}$	98,215.14	128.66	11.77	139.68	69.22	140.71
$t_{10}$	98,215.14	128.66	11.77	139.68	69.22	140.71
$t_{p}$	90,056.57	140.32	10.04	163.75	61.49	158.41

**Table 3:** PRE of different estimators with respect to $t_{0}$ for the simulated population.

Estimator	$t_{0}$	$t_{r}$	$t_{1}$	$t_{2}$	$t_{3}$	$t_{4}$	$t_{5}$	$t_{6}$	$t_{7}$	$t_{8}$	$t_{9}$	$t_{10}$	$t_{p}$
PRE	100.00	138.47	149.81	149.81	61.76	68.52	85.94	149.81	149.81	149.81	149.81	149.81	172.68

## Conclusion

In this manuscript, we have introduced a new class of estimators of $\overline{Y}$ under imputation method. The bias and the MSE of the introduced family have been studied for an approximation of degree one. Through theoretical comparison with competing estimators using imputation techniques, efficiency requirements over competing estimators are produced for the suggested estimator. Along with a simulated population, three actual natural populations are used to confirm these efficiency criteria of the presented estimators. It has been found that the suggested estimators is having least MSE in all three real populations and has heighest PRE for all real and simulated populations. Thus it is evident that the introduced estimator is the most efficient among the class of all estimators of $\overline{Y}$ in competition under the imputation methods. As the proposed estimator is most efficient, therefore it is recommended for use in different ares of applications including Agricultural Sciences, Biological Sciences, Commerce, Engineering, Economics, Fishries, Medical Science, Social Science and other areas of applications. For instance, as certain experimental units must be discarded during the experiment, in therapeutic or life-saving drug testing studies. In a manner similar to this, diseases, livestock grazing, or other natural calamities kill crops during agricultural experiments.

## Data Availability

All relevant data is within the manuscript.
